# Plasma Exosomes and Improvements in Endothelial Function by Angiotensin 2 Type 1 Receptor or Cyclooxygenase 2 Blockade following Intermittent Hypoxia

**DOI:** 10.3389/fneur.2017.00709

**Published:** 2017-12-22

**Authors:** Abdelnaby Khalyfa, Nina Youssefnia, Glen E. Foster, Andrew E. Beaudin, Zhuanghong Qiao, Vincent Pialoux, Matiram Pun, Patrick J. Hanly, Leila Kheirandish-Gozal, Marc J. Poulin, David Gozal

**Affiliations:** ^1^Section of Pediatric Sleep Medicine, Department of Pediatrics, Pritzker School of Medicine, Biological Sciences Division, University of Chicago, Chicago, IL, United States; ^2^Department of Physiology and Pharmacology, Cumming School of Medicine, University of Calgary, Calgary, AB, Canada; ^3^Cumming School of Medicine, Hotchkiss Brain Institute, University of Calgary, Calgary, AB, Canada; ^4^Department of Medicine, Cumming School of Medicine, University of Calgary, Calgary, AB, Canada; ^5^Department of Clinical Neurosciences, Cumming School of Medicine, University of Calgary, Calgary, AB, Canada; ^6^Cumming School of Medicine, Libin Cardiovascular Institute of Alberta, University of Calgary, Calgary, AB, Canada; ^7^Faculty of Kinesiology, University of Calgary, Calgary, AB, Canada

**Keywords:** cardiovascular disease, intermittent hypoxia, sleep apnea, experimental human model, endothelium, exosomes, angiotensin receptor, cyclooxygenase 2

## Abstract

Intermittent hypoxia (IH) is associated with increased endothelial dysfunction and cardiovascular disorders. Exosomes released in biological fluids may act as vehicles for propagating such damage, modifying the functional phenotype of endothelial cells. Drug interventions, however, may provide protection for the endothelium, in spite of exosomal activity. Using an experimental human model of IH, we investigated whether the beneficial effects of two drugs, celecoxib (CEL) and losartan (LOS), on IH-induced vascular dysfunction was mediated *via* exosomes or independent of IH-induced exosomal cargo alterations. We hypothesized that the beneficial effects of CEL and LOS on IH-induced vascular dysfunction would be mediated *via* modifications of exosomal properties by the drugs, rather than by direct effects of the drugs on the endothelium. Ten male volunteers were exposed to IH (single exposure of 6 h) while receiving LOS, CEL, or placebo (P) for 4 days before IH exposures, and plasma samples were obtained from which exosomes were isolated, and incubated with naïve human endothelial cell cultures either not treated or pretreated with LOS, CEL, or P. Functional reporter assays (monolayer impedance, monocyte adhesion, and eNOS phosphorylation) revealed that the degree of exosome-induced endothelial dysfunction was similar among IH-exposed subjects independent of drug treatment. However, pretreatment of naïve endothelial cells with LOS or CEL before addition of exosomes from IH-exposed subjects afforded significant protection. Thus, the cardiovascular protective impact of LOS and CEL appears to be mediated by their direct effects on endothelial cells, rather than *via* modulation of exosomal cargo.

## Introduction

Obstructive sleep apnea (OSA) is a highly prevalent chronic medical condition associated with increased cardiovascular morbidity and mortality (1, 2). OSA is characterized by repeated apneas during sleep due to recurring collapse of the pharynx, resulting in intermittent hypoxia (IH). In healthy humans, exposure to experimental IH even for short periods of time lasting days to weeks induces disturbances in vascular regulation, the latter manifesting as increased resting systemic inflammation and blood pressure, as well as endothelial dysfunction (3, 4).

Cyclooxygenase isoenzyme COX-2 serves as an important mediator of inflammatory responses and has been mechanistically implicated in IH-induced deleterious effects in both cardiovascular and central nervous systems (5). Similarly, alterations in angiotensin II activity have been suggested as a critical mechanism underlying blood pressure elevations and vascular dysfunction in the context of enhanced oxidative stress (6). In this setting, drugs such as celecoxib (CEL) and losartan (LOS), which operate as a selective COX-2 inhibitor and as a selective angiotensin II receptor 1 antagonist, respectively, have been shown to attenuate the cardiovascular dysfunction induced by experimental IH in humans (7, 8).

Exosomes are ubiquitous microvesicles originating from different subcellular compartments whose generation, content, and release are highly regulated processes within the cellular machinery. In recent years, it has become apparent that exosomes play significant roles as mediators of intercellular communication in the cardiovascular system and are pathophysiologically involved in processes leading to endothelial dysfunction and atherosclerosis, usually through miRNA transfer to target cells (9). We have recently shown that plasma-derived exosomes from otherwise healthy young experimental subjects exposed to 4 days of IH adversely disrupt endothelial cell function *in vitro*, and that such properties are also reflected in their differential miRNA cargo expression (10). In this study, we aimed to elucidate whether the beneficial effects of LOS or CEL on IH-induced vascular dysfunction (7, 8, 11) are mediated *via* direct modifications of exosomal properties by the drugs, whether these drugs protect the endothelium directly but do not alter exosomal properties, or whether the beneficial effects reflect a combination thereof.

## Materials and Methods

Twenty healthy male subjects participated in the studies (*n* = 10 for each drug) as previously described (7, 8, 11). Briefly, their mean (±SD) age was 29.3 ± 1.7 years, and their BMI was 25.6 ± 0.4 kg/m^2^. The research study was approved by the Conjoint Health Research Ethics Board at the University of Calgary, and written informed consent was obtained from each subject before participating in the study. In addition, the study was also approved by the Human Subject committee at the University of Chicago (protocol # 10-702-A-CR004). The experimental human model of IH, along with the details of the protocol has been previously described (7). Briefly, IH was induced by alternating between 1 min of hypoxia and 1 min of normoxia for 6 h, thereby mimicking an oxygen desaturation index of 30 events/h (i.e., moderate-to-severe OSA). Subjects wore a full face respiratory mask (Mirage NV Full Face Mask Series 2; Resmed, NSW, Australia) connected to a two-way non re-breathing valve with a 30 cm long mixing tube connected to the inspired side. IH was performed in a custom built hypoxic chamber in which the gas composition was maintained at a level sufficient to decrease P_ET_O_2_ to 45.0 mmHg within 60 s. Periods of normoxia were elicited by delivering 100% oxygen to the subject through the mixing tube connected to the mask with the flow rate adjusted to increase P_ET_O_2_ to 88.0 mmHg within 60 s. When oxygen flow through the mixing tube was 0, the subject breathed the gas composition of the hypoxic chamber. To maintain P_ET_CO_2_ at normal levels 100% CO_2_ was delivered through the mixing tube during hypoxic periods. Respired gas was sampled from a nasal cannula and continuously analyzed by a dual oxygen and carbon dioxide analyzer (NormocapOxy, Datex-Ohmeda, Louisville, CO, USA) for PO_2_ and PCO_2_ during all experimental sessions. The identical experimental setup was used for subjects being randomly assigned to receive either drug or placebo (P).

**Figure 1:**
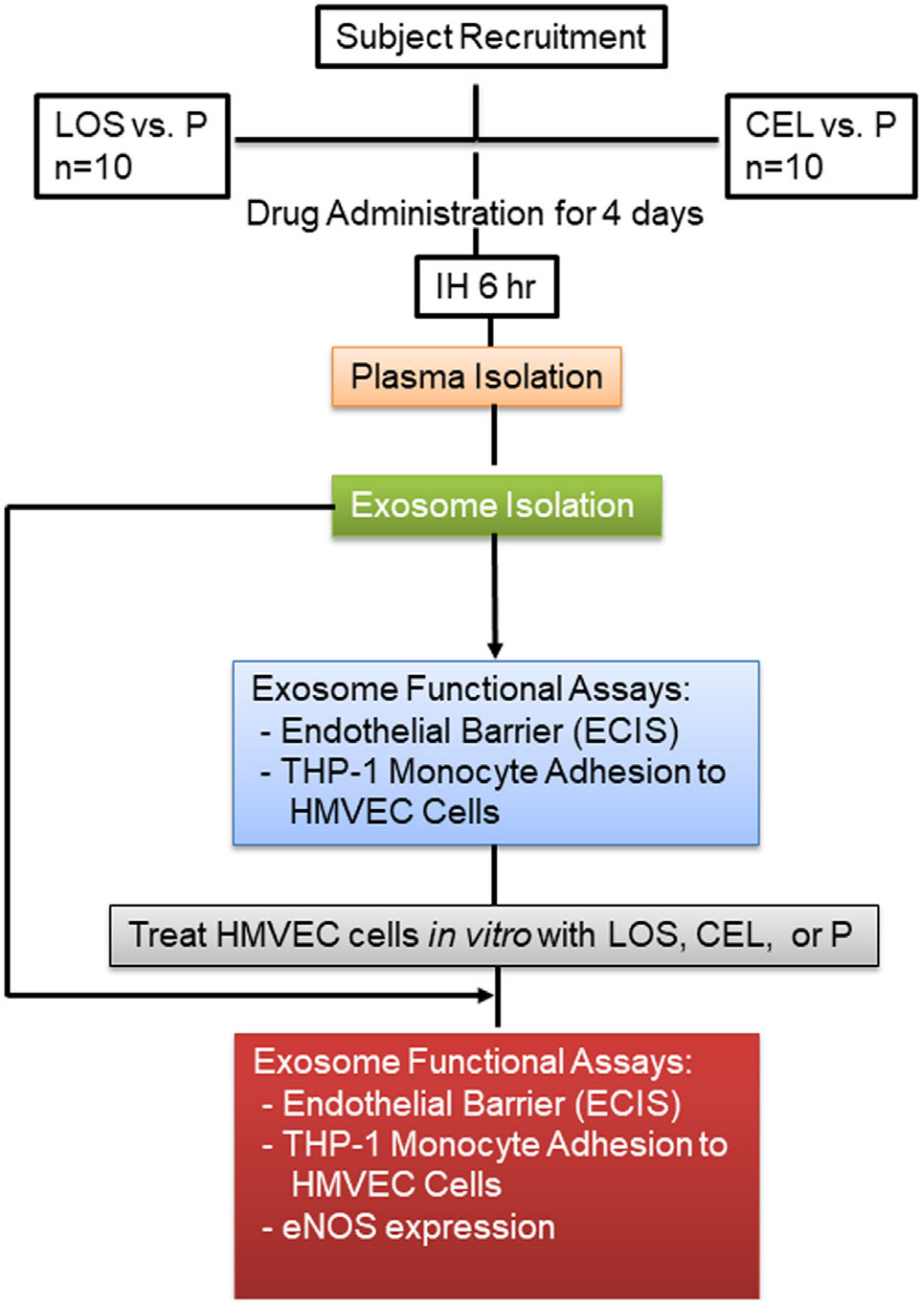
Schema illustrating subject recruitment, drug used, and data analysis. Plasma was isolated from each subject, and equal volume for each sample was used to isolate circulating exosomes.

In one set of experiments, all 10 subjects were treated with LOS or P in random order while exposed to IH, with both subjects and experimenters being blinded as to the treatment being administered. All subjects were submitted to three experimental protocols [IH + losartan (IH-LOS), IH + placebo (IH-P) and sham IH (room air)] separated from each other by at least 1 week, and occurring in random order at the same time of day (i.e., between 09:00 and 15:00 h) (7, 8). In addition, to avoid differences in the consumption of exogenous antioxidants, subjects were asked to follow the same diet for 3 days preceding each experimental protocol. Dietary compliance was monitored by food diaries and urinary sodium excretion. All protocols involved a 6 h exposure to either IH or sham IH. For the IH + LOS protocol, subjects were administered 25 mg of LOS on day 1, 50 mg on day 2, and 100 mg on days 3 and 4. Preceding the IH + P protocol, all subjects were administered P tablets for four consecutive days in the morning before IH exposure. Venous blood samples were collected into EDTA tubes from the antecubital vein at the beginning and immediately at the end of each experimental protocol (less than 10 min after the cessation of IH). The plasma was obtained by centrifugation of the samples at 1,000 × *g* for 10 min at 4°C. Plasma was separated into aliquots and frozen at −80°C until assays. In a second set of experiments, the same approach was employed in the same set of 10 subjects at a different time, with the exception that CEL was administered as follows: For 4 days preceding each IH exposure, participants was given either P (p.o. three times daily at 08:00, 14:00, and 20:00 h), or CEL (200 mg p.o. twice daily at 08:00 and 20:00 h). To maintain the double-blinded nature of the study, a P tablet was included in the CEL group as the second tablet. On experimental day of IH exposures, the dosage regimen was maintained through to the end of the exposure (12). A schematic diagram of the experimental design is shown in Figure 1.

### Exosomes Isolation

As in previous studies (13–15), exosomes were isolated from plasma using the Total Exosome Isolation Kit according to the manufacturer’s protocol (Life Technologies, Carlsbad, CA, USA), and further characterized in accordance with the consensus guidelines of the International Society for Extracellular Vesicles (16). Briefly, plasma was centrifuged at 2,000 × *g* for 20 min to remove cell/debris. The supernatants were collected, and 0.2 volume of the Total Exosome Isolation Reagent was added. The mixtures were incubated at 4°C for 30 min followed by centrifugation at 10,000 × *g* for 10 min, and pellets were solubilized in 1× phosphate-buffered saline (PBS).

### Exosomes Uptake and Flow Cytometry

For exosome uptake assessment, total purified exosomes were labeled using PKH67GL (Sigma-Aldrich, St. Louis, MO, USA), and images were acquired using confocal microscopy. Exosomes were labeled for 10 min at 37°C, and the mixtures were precipitated with ExoQuick-TC reagents (System Biosciences) and further incubated on ice for 30 min. The mixtures were centrifuged at 4°C at 13,000 rpm for 4 min. The pellets were suspended in 1× PBS buffer, and the labeled exosomes were delivered to confluent coverslips of human microvascular endothelial cells (HMVEC; Lonza catalog #: CC-2543; Allendale, NJ, USA) for 6 h in a cell culture incubator at 37°C. Labeled PKH67-Green color was then monitored for delivery into target cells using a Leica SP5 Tandem Scanner Spectral 2-photon confocal microscope (Leica Microsystems, Inc., Buffalo Grove, IL, USA) with a 63× oil-immersion lens. As a negative control, PKH67-Green was prepared with all reagents, but without exosomes to monitor unincorporated dyes carried over after the centrifugation. The nucleus of the cells was visualized by staining with Hoechst 33342 (Sigma-Aldrich, St. Louis, MO, USA) at a concentration of 1 µg/mL in PBS at room temperature for 5 min.

For flow cytometry, purified exosomes were incubated with Exo-Flow™ kits (System Biosciences, Mountain View, CA, USA) to analyze for selective subpopulations of exosome surface marker using FACS analysis (FACSCalibur, BD Biosciences, San Jose, CA, USA). Briefly, purified exosomes were incubated with magnetic beads 9.1 µm with different exosome markers including: CD9 (for exosomes formation and secretion), CD31 (for exosomes maturation and target cell binding), HLA-G (immune modulation, energy and priming), and Rab5a (exosomes biogenesis, secretion, and downstream cell fusion). Exosomes and magnetic streptavidin beads were incubated overnight at 4°C according to the manufacturer’s recommendations. Negative control experiments were also carried out with all the reagents and beads but without exosomes. Thirty thousand events were acquired and computed, and data were analyzed using FlowJo Software (Tree Star, Inc., Ashland, OR, USA) (see Figure S1 in Supplementary Material).

### Endothelial Cell Culture

Human microvascular endothelial cells were purchased from Lonza (catalog #: CC-2543; Allendale, NJ, USA). Cells were cultured in endothelial growth medium (EGM™-2MV BulletKit™; Clonetics) supplemented with 5% fetal bovine serum (FBS; Clonetics) and incubated at 37°C and 5% CO_2_ in cell culture incubator. For continuous passaging, the cells were trypsinized and dissociated, then were centrifuged at 220 × *g* for 7 min, diluted, and re-plated at the appropriate densities. All cells were used before passage 4.

### Electric Cell-Substrate Impedance Sensing (ECIS)

Endothelial cells were grown in DMEM media containing 2% FBS for 24 h. Cells were seeded (30,000 cells) and grown to confluence into ECIS arrays as a single confluent monolayer. Exosomes were added in duplicate wells and placed into the ECIS instrument^1^ for continuous monitoring up to 72 h. The ECIS array enables assessment of morphological and structural cell changes, cell locomotion and displacement, and other behaviors mediated by activity within the cell’s cytoskeleton. ECIS uses 250 µm diameter gold-film electrodes deposited on the bottom of cell culture dishes and measures the electrode impedance. As cultured cells attach and spread on the electrode surface, impedance is altered and serves as a measure for disruption of the endothelial cellular junction (17). This method is based on measuring non-invasively the frequency-dependent electrical impedance of cell-covered gold-film electrodes along the time course of the experiment.

For initial experiments equivalent numbers of plasma exosomes from CEL, LOS, and corresponding P controls (CTL) were added to the wells and ECIS was continuously monitored. In additional experiment, either CEL or LOS was added to experimental wells, leaving partnered control wells treated with vehicle, and incubated for 6 h. Exosomes were then added in duplicate wells for continuous monitoring up to 24 h. To determine optimal drug concentrations for use *in vitro*, ECIS arrays were also utilized and concentration of drugs was determined for use in preliminary experiments.

### Drug Preparation and Experimental Protocol

Celecoxib (PZ0008-5MG; Sigma-Aldrich, St. Louis, MO, USA) stock was reconstituted in DMSO and prepared fresh from stock for each experimental run to avoid variability due to thawing and refreezing. From the 50 mM stock, CEL was prepared at a final concentration of 5 µM in EGM™-2MV BulletKit™ supplemented with 2% FBS. LOS (cat. # PHR1602, Sigma-Aldrich, St. Louis, MO, USA) stock was prepared at concentration of 1,000 mM in EGM™-2MV BulletKit™ supplemented with 10% FBS, again with fresh stock utilized for each run. From the 1,000 mM stock, LOS was prepared at a final concentration of 50 µM by dilution in EGM™-2MV BulletKit™ without FBS.

For experimental conditions, once endothelial cells reached confluence as a single monolayer in EGM™-2MV BulletKit™, medium was changed to EGM™-2MV BulletKit™ supplemented with 2% FBS, after which the drug (40 µL CEL; 25 µL LOS) was added and incubated for 6 h. Exosomes from IH untreated subjects were then added to wells and incubated for 24 h. All runs utilized the same four experimental conditions, namely, control, control plus drug, IH, and IH plus drug. A dose–response for each drug was prepared in advance to identify optimal concentrations.

### Monocyte Adhesion Assays

Human THP-1 NucLight™ Red Cells were supplied in 1 mL cryopreserved vials (1 × 10^6^ cells/mL in 90% FBS and 10% DMSO) containing a stable population of human monocytic cells expressing the NucLight™ Red fluorescent protein, with expression in the latter being restricted to the nucleus. Cells were a kind gift from Essen Bioscience company.^2^ Briefly, cells were grown in Recommended Media and Components in RPMI (Life Technologies, Carlsbad, CA, USA), 10% FBS (Life Technologies, Carlsbad, CA, USA), 0.05 mM β -mercaptoethanol (Life Technologies, Carlsbad, CA, USA), 1% Pen/Strep (cat. # 15140 Gibco/Life Technologies), and 0.5 µg/mL Puromycin (cat. # A11138-03 Gibco/Life Technologies) at 37°C incubator, 5% CO_2_. Cells were regularly checked for Red Color under fluorescent microscope. Following incubation with drug and exosomes, as described earlier, THP-1 red monocytes (4 × 10^6^) were suspended in EGM™-2MV BulletKit™ supplemented with 2% FBS and were added to the cell culture (100 μL/well) for 30 min at 37°C, and then washed with cold PBS two times to remove unbound THP-1 red monocytes and identify adhered monocytes. Cells were then fixed with 4% paraformaldehyde (PFA) for 15 min at room temperature. PFA was then removed, and remaining cells in culture plates were suspended in cold PBS. Adherent monocytes were counted on a fluorescent microscope by an investigator who was blinded to the experimental conditions.

### Western Blot

Human microvascular endothelial cells were grown in six-well plates as mentioned earlier. After 2 days, the cells were confluent and treated with drugs either CEL (PZ0008-5MG; Sigma-Aldrich, St. Louis, MO, USA) or LOS (PHR1602, Sigma-Aldrich, St. Louis, MO, USA) for 6 h. Cells without drugs were also included as negative CTL. Exosomes derived from subjects exposed to IH were added in the presence of and without drugs for 24 h in media 2% FBS. Adherent cells were washed with warm PBS, and 2% sodium dodecyl sulfate solution was added to each well (120 μL/well) and left on shaker for 20 min to collect and lyse cell contents. Protein concentrations were evaluated using the BCA kit (Life Technologies). Equal amounts of total proteins from individual strain were electrophoresed using sodium dodecyl sulfate-polyacrylamide gel electrophoresis [10% and transferred into nitrocellulose membrane (Millipore, Billerica, MA, USA)]. After transfer, membranes were incubated in blocking buffer (5% nonfat dry milk in TBST) for 1 h at room temperature. The membranes were incubated with phospho-eNOS (Ser1177, C9C3; p-eNOS) rabbit mAb antibody (Cell Signaling Technology, Danvers, MA, USA; 1:1,000 dilution overnight at 4°C), then washed three times for 10 min each with 25 mm Tris, pH 7.4, 3.0 mm KCl, 140 mm NaCl, and 0.05% Tween-20 (TBST), incubated with anti-rabbit immunoglobulin G:HRP-linked antibody (Cell Signaling Technology; 1:2,000 dilution in blocking buffer with gentle agitation for 1 h at room temperature), and finally immunoreactive bands were visualized using an enhanced chemiluminescence detection system (Chemidoc XRS+; Bio-Rad, Hercules, CA, USA), and quantified by the Image Lab software (Bio-Rad, Hercules, CA, USA). The intensity of p-eNOS was normalized to β-actin as a control.

### Data Analysis

Data are expressed as means ± SE unless otherwise indicated. Statistical analyses were performed using SPSS statistical software (version 21.0; Chicago, IL, USA). Comparisons between exosome-treated and untreated cells were performed using *t*-tests or one-way analysis of variance with Bonferroni correction for multiple comparison tests. A value of *p* < 0.05 was considered a statistically significant difference unless otherwise indicated.

## Results and Discussion

### Exosomes Uptake

**Figure 2:**
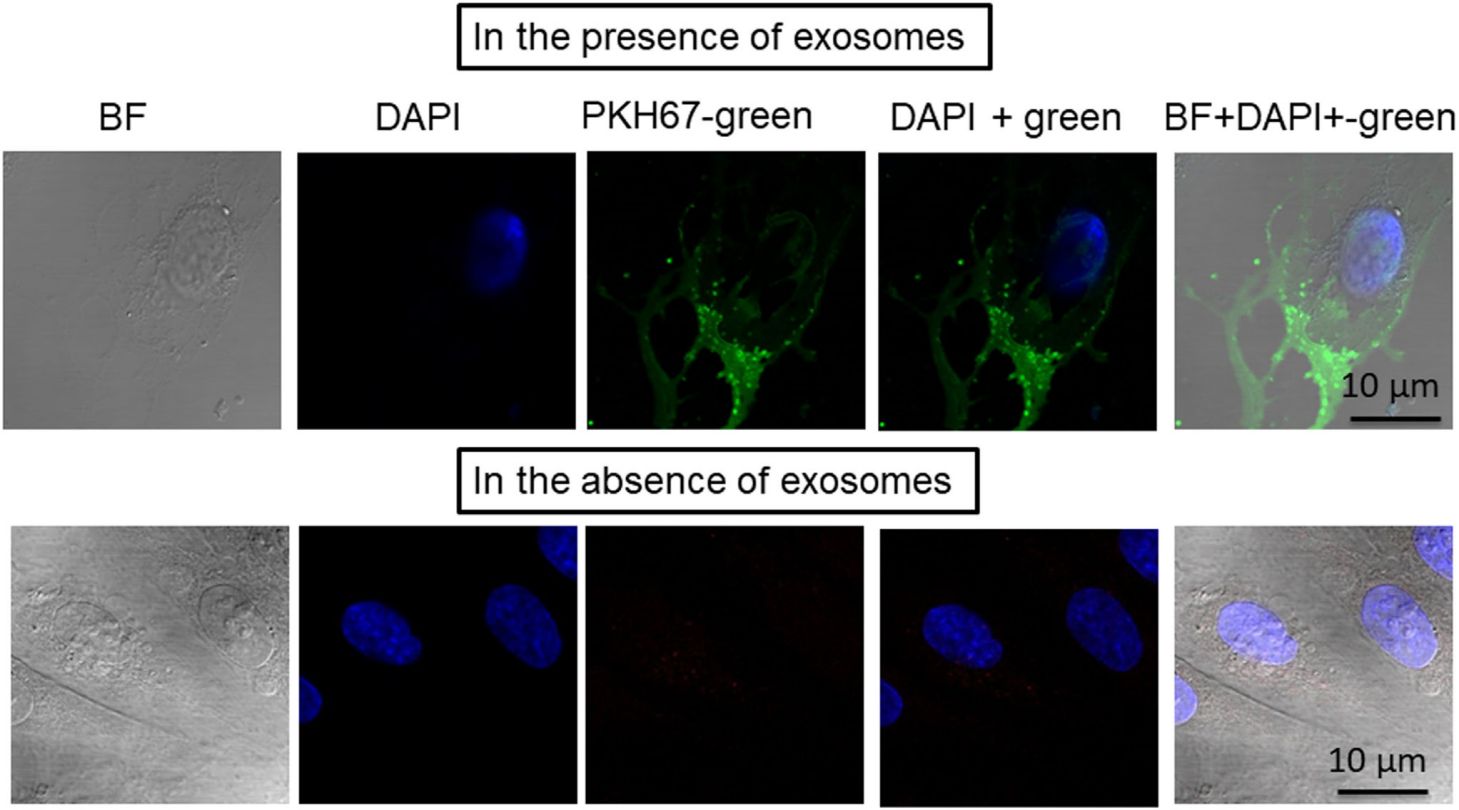
Confocal microscope images illustrating exosome uptake into human endothelial cells. Exosomes were isolated from plasma of subjects exposed to intermittent hypoxia and labeled with the PKH67-Green Fluorescent Cell Linker Kit. Human endothelial cells were grown on coverslips for 24 h, and the labeled exosomes with PKH67 were added to the cells for 6 h at 37°C. Cells were washed and stained with nuclei (blue) stained with DAPI. Exosome uptake was measured using confocal laser scanning microscopy at 490 nm excitation and 502 nm emissions. *n* = 6, scale bar in 10 µm. As controls, no exosomes were used but PKH67 was added.

To test whether exosomes isolated from human plasma were incorporated into human naïve endothelial cells, the culture media of the cells was supplemented individually with PKH67-labeled exosomes (Figure 2). The PKH67 signal was observed in the lipid cell membrane of cells grown in medium supplemented with PKH67-labeled exosomes, whereas no signal was observed in cells grown in medium supplemented without exosomes that to which PKH67 was also added (Figure 2). The imaging approaches suggested that circulating exosomes derived from subjects exposed to IH contain active microvesicles that are effectively delivered to endothelial cells and are incorporated into these cells. Exosomes are known to transport their cargo, including proteins, mRNAs, and miRNAs from parental cells to recipient cells. In addition, exosomes are currently believed to play a role in regulating physiology and pathophysiology by mediating cell–cell communication (18, 19). Aside from validation of the quality of purified exosome samples using known exosomal markers (see Figure S1 in Supplementary Material), it is also important to determine that exosomes are bioactive and can be taken up after isolation procedures (Figure 2), as a correlate that isolated exosomes have retained appropriate biological functions (20). However, how exosomes interact with recipient cells and how exosomes are sorted after entry into these cells remain unclear (21, 22).

As with any subcellular organelle, it is important to determine unique markers specific to exosomes to aid in the validation of purified exosome samples. In this study, we used different exosome markers including: tetraspanins (CD9; exosome formation and secretion), targeting/adhesion (CD31; exosome maturation and target cell binding), antigen presentation (HLA-G; immune modulation), and membrane transport (Rab5b; exosome biogenesis, secretion and cell fusion), as shown in Figure S1 in Supplementary Material.

### ECIS Experiments

**Figure 3:**
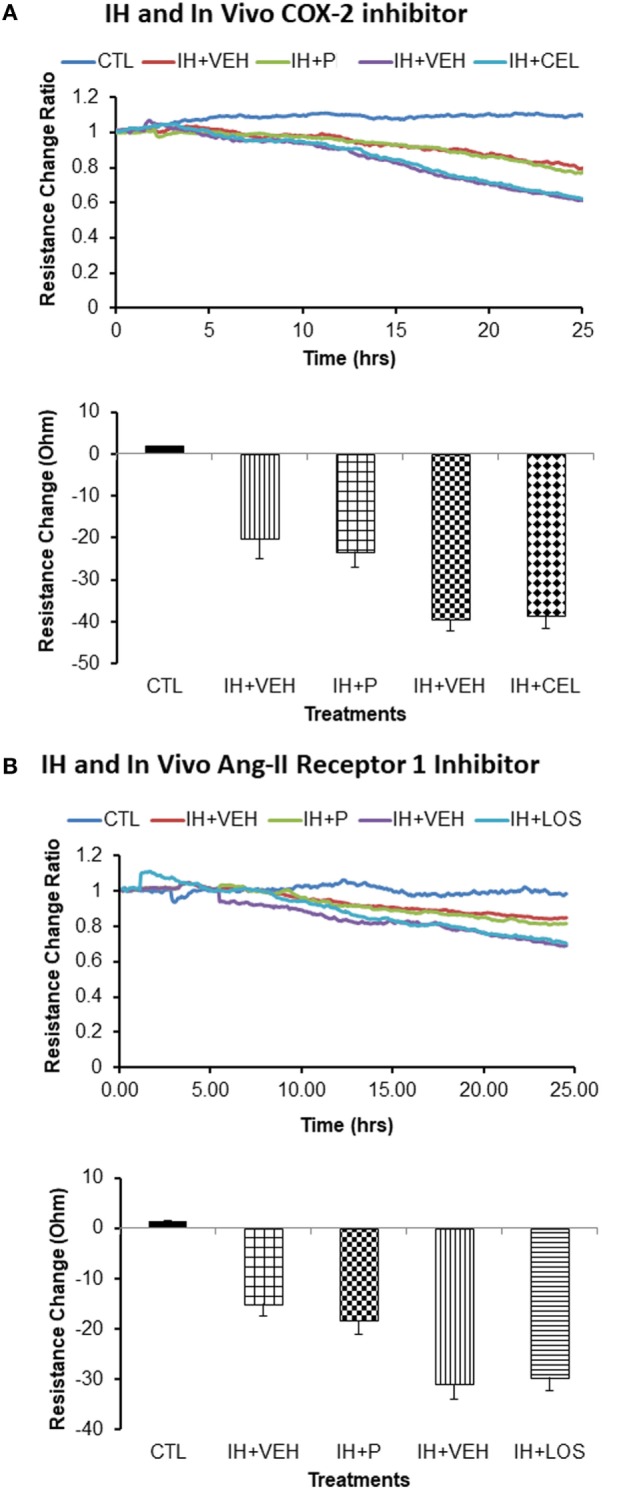

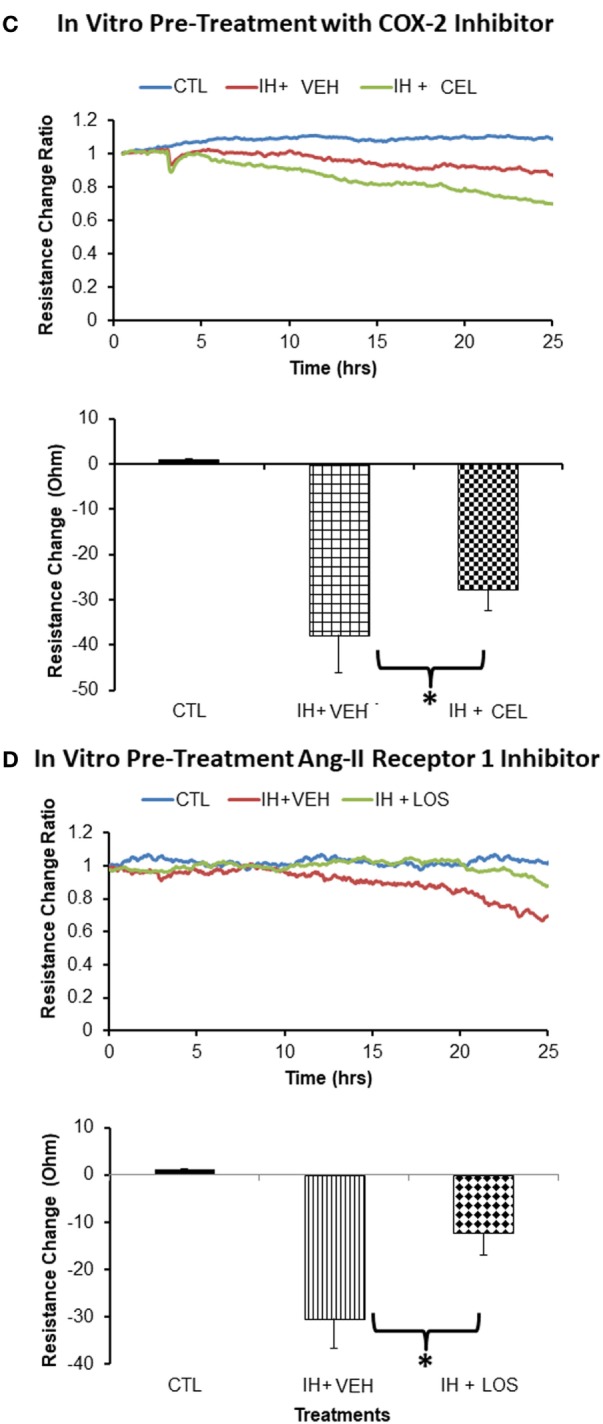
Effect of plasma-derived exosomes on electric cell-substrate impedance sensing (ECIS) in human microvascular endothelial cells. Plasma-derived exosomes from subjects exposed to intermittent hypoxia (IH) and treated with celecoxib (CEL) induce similar cellular barrier disruption as identified *via* impedance sensing technology (ECIS) in an endothelial cell monolayer as when subjects are exposed to IH but received placebo (P), CEL **(A)** or losartan (LOS) **(B)**. Similar findings emerged for LOS *in vivo* treatment, whereby exosomes from IH + losartan (IH + LOS) subjects did not alter the disruption of the endothelial cell barrier as measured by ECIS compared with P-treated conditions. However, *in vitro* pretreatment of endothelial cells with either CEL **(C)** or LOS **(D)** afforded significant protection (*p < 0.01). Graphs show the average resistance changes (normalized to time = 0) from each group before and after treatments. Data are presented as mean ± SD (*n* = 10/group).

Treatment of naïve endothelial cells with plasma-derived exosomes from subjects exposed to IH elicited marked reductions in impedance independently from the drug treatment. Indeed, there were no significant differences in endothelial cell tight junction disruption between CEL and corresponding P-derived exosomes (Figure 3A) or between plasma exosomes from subjects treated with LOS or corresponding CTL (Figure 3B). In contrast, when endothelial cells were pretreated *in vitro* with either CEL (Figure 3C) or LOS (Figure 3D), subsequent addition of plasma exosomes from IH-exposed subjects resulted in attenuated effects on monolayer resistance when compared with vehicle-pretreated wells (*p* < 0.01 drug vs control; *n* = 10).

### IH-Derived Exosomes and THP-1 Red Monocyte–Endothelial Cell Adhesion

**Figure 4:**
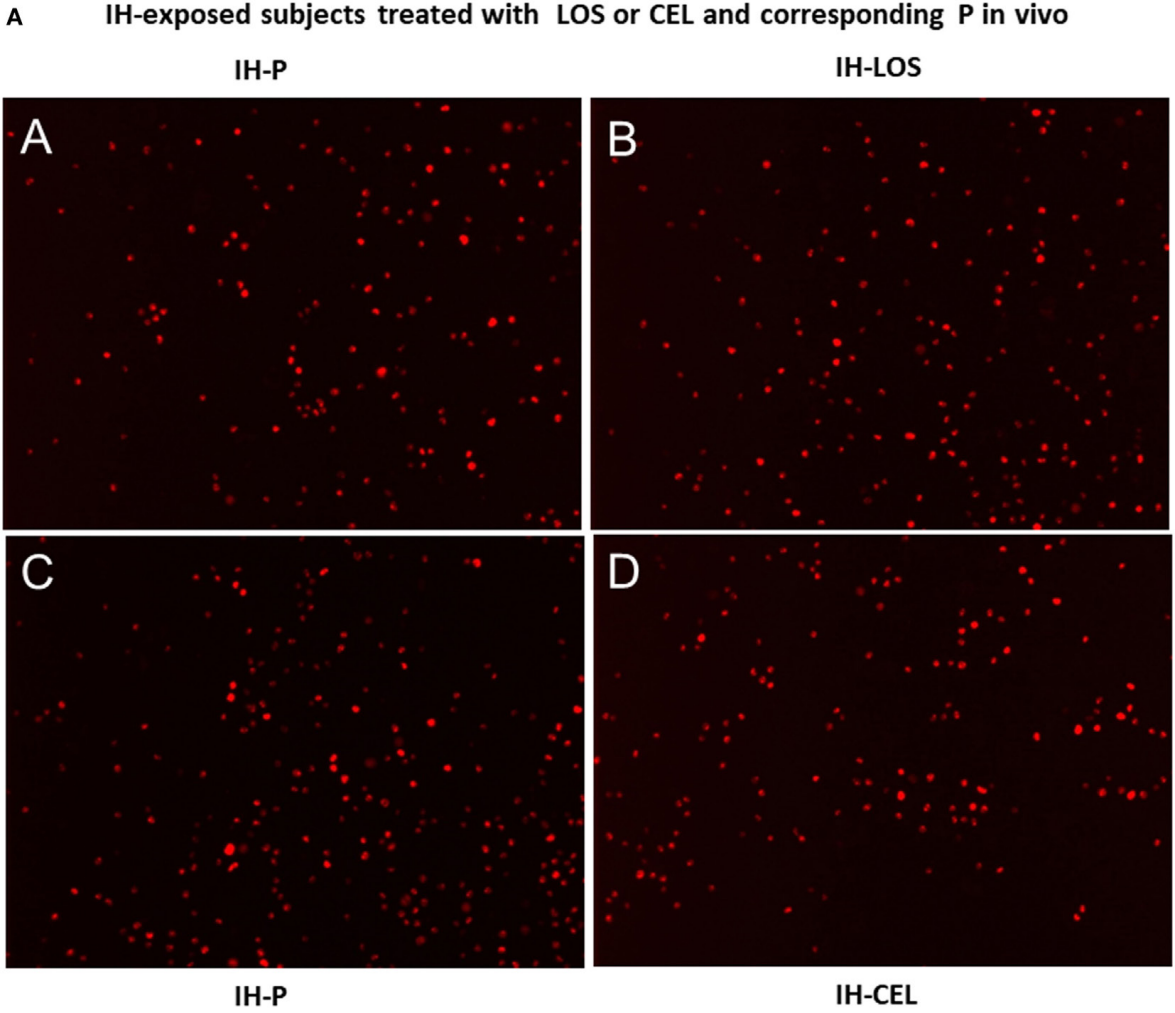

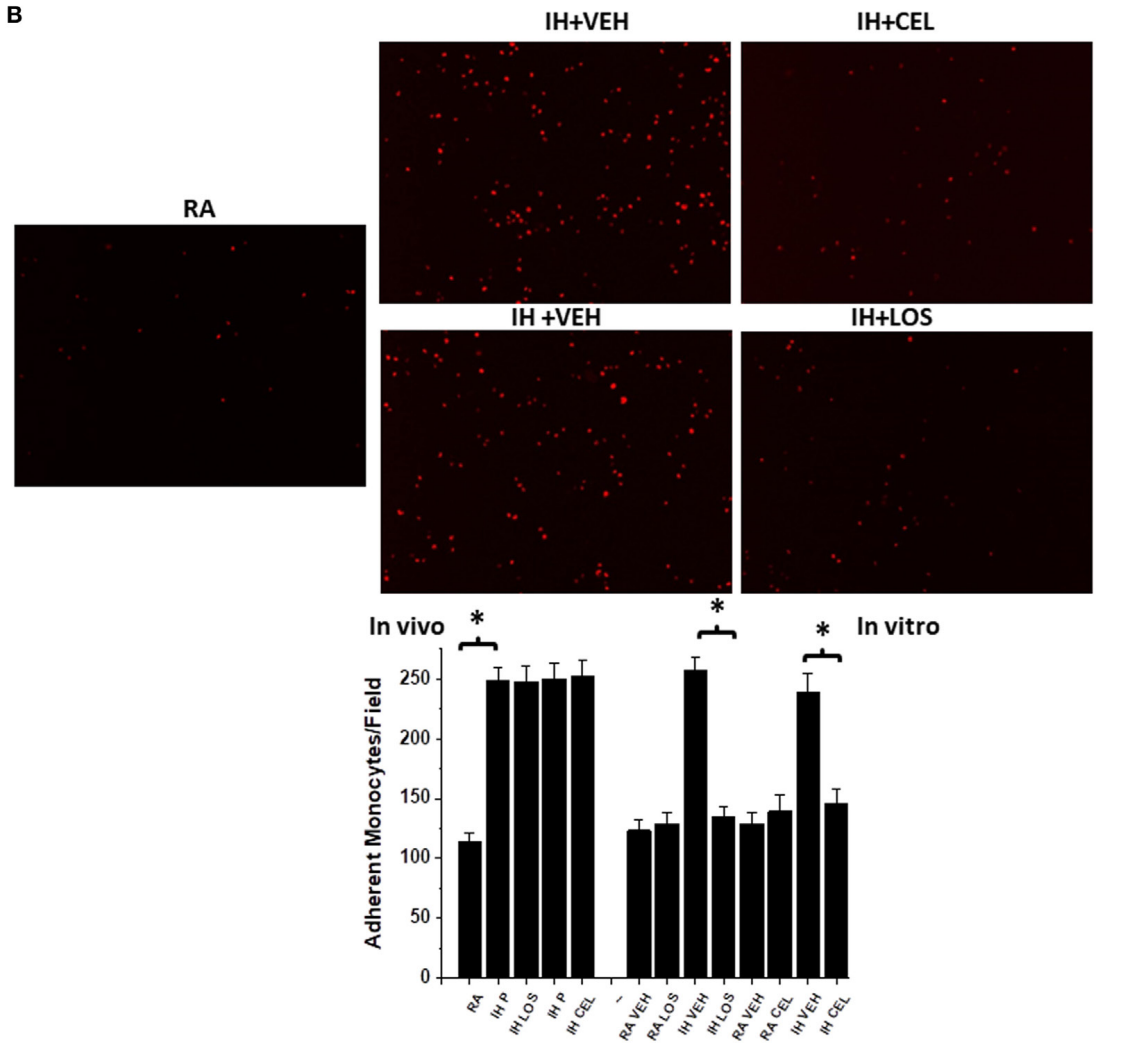
Effects of exosomes-derived from adult subjects exposed to intermittent hypoxia (IH) for days on adherence of human monocytes stably transfected with red fluorescent protein (THP-1 Red) in the presence and absence of drugs [celecoxib (CEL) or losartan (LOS)]. Human endothelial cells were grown in six-well plates until reaching confluence, and exosomes were added for 24 h, followed by addition of THP-1 human monocytes red (4 × 10^6^) for 30 min, and then cells were washed to reveal adherent monocytes. **(A)** (A,B) are representative images of adherent monocytes after exosomes from IH-exposed subjects treated with placebo (P) or LOS were added, while (C,D) show similar experiments for P or CEL treatments *in vivo*. No significant differences emerged between P and treatment *in vivo*, but all conditions were significantly different from normoxia [room air (RA); **(B)** lower panel]. **(B)** Representative images of monocyte adherent cells after endothelial cells were exosomes pretreated with drugs or vehicle. Both CEL and LOS pretreated endothelial cells attract less THP-1 red monocytes *in vitro* and restore the number of adherent monocytes to RA levels (see bottom panel for summary of all findings). In each image of experiment run, six different fields per subject were counted and averaged for each subject (*n* = 10 subjects/group).

Initial experiments using plasma exosomes from IH-exposed subjects treated with LOS, CEL, or corresponding control conditions failed to reveal any significant differences among these conditions, and collectively showed marked and similar increases in THP-1 monocyte adherence compared with untreated endothelial cells (Figure 4A). To further elucidate whether pretreatment of endothelial cells with drug could dampen monocyte adhesion, the four experimental conditions employed in these experiments [i.e., *in vitro* HMVEC exposed to control (no exosomes), control pretreated with drug, plasma exosomes from IH-exposed subjects pretreated with vehicle, and plasma exosomes from IH-exposed subjects pretreated with drug] were implemented and followed by addition of THP-1 red fluorescent monocytes. Compared with all other three conditions, endothelial cells incubated with IH-exposed exosomes and pretreated with vehicle showed significant increases in THP-1 monocyte adhesion, and such effect was markedly attenuated when endothelial cells were pretreated with either LOS or CEL (Figure 4B).

### Effects of Exosomes on eNOS Phosphorylation *In Vitro*

**Figure 5:**
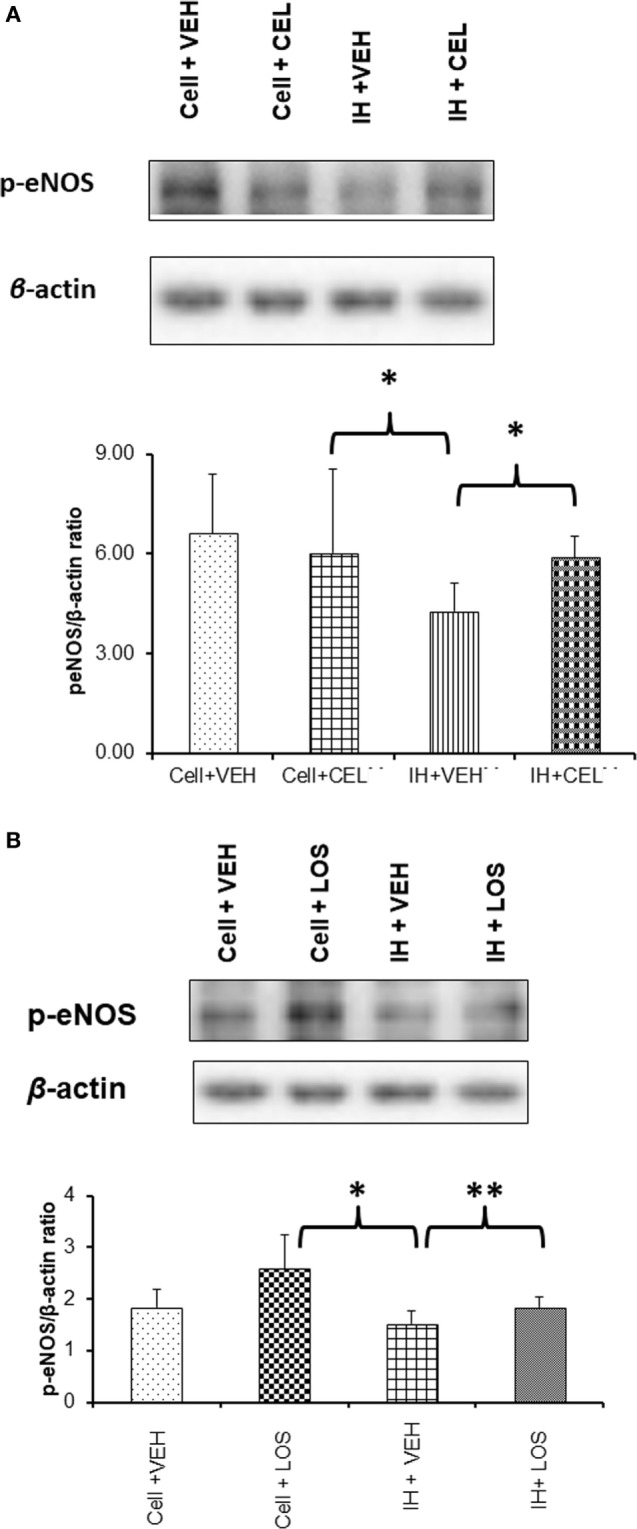
Western blot analysis for phosphorylated-eNOS (p-eNOS) at serine residue 1133 in human endothelial cells treated with and without drugs [celecoxib (CEL) or losartan (LOS)]. Cells were grown in six-well plates at 37°C for 48 h in endothelial growth media (EGM™-2MV BulletKit™) and then treated with CEL or LOS for 6 h in the same media supplemented with 2% fetal bovine serum. Plasma-derived exosomes from adult subjects exposed to intermittent hypoxia (IH) were applied to the endothelial cells for 24 h, after which proteins were isolated and subjected to western blots probed with p-eNOS antibody and β-actin. **(A)** A representative western blot for endothelial cells alone or treated with CEL, or cells treated with exosomes from IH-exposed subjects and either pretreated with vehicle or CEL. In the bottom panel, summary of densitometry assessments for p-eNOS immunoreactivity is shown (*p < 0.01, vehicle pretreatment followed by IH exosomes vs. no exosomes, and *p* < 0.01 CEL vs. VEH pretreatment followed by IH exosomes; *n* = 8/group). **(B)** Similar experiments as in panel **(A)**, except that LOS was the drug used for pretreatment of endothelial cells *in vitro* (*p < 0.01, vehicle pretreatment followed by IH exosomes vs. no exosomes, and **p < 0.04 LOS vs. VEH pretreatment followed by IH exosomes; *n* = 8/group).

*In vitro* administration of exosomes from IH-exposed subjects induced significant reductions in eNOS phosphorylation at Ser 1133 (Figure 5). However, after pretreatment of endothelial cells with either CEL or LOS *in vitro*, administration of plasma-derived exosomes from IH-exposed subjects did not induce as significant reductions eNOS phosphorylation, with CEL preserving eNOS phosphorylation state at untreated levels (Figure 5A), and LOS significantly attenuating the effect of IH-derived exosomes (Figure 5B).

In this study, we show that plasma-derived exosomes from IH-exposed experimental subjects treated with P or LOS or CEL elicit similar deleterious effects on several reporter assays of endothelial cell function, suggesting that treatment with these compounds does not alter the functional properties of the circulating exosomes. Such findings provide a more detailed perspective on the previously reported beneficial effects of both CEL and LOS on the vasculature (7, 8, 11), and raise the possibility that, contrary to our initial hypothesis, the protective effects of either CEL or LOS are not by modification of exosomal cargo and corresponding functional properties *per se*, but rather *via* a direct effect on the target cells, i.e., the endothelium. Indeed, pretreatment of naïve endothelial cells with either LOS or CEL led to significant attenuations in the adverse effects of plasma-derived exosomes from human subjects exposed to IH. Thus, in the context of elucidating the role of exosomes in the processes leading to the cardiovascular dysfunction associated with IH exposures mimicking sleep apnea, it became apparent that treatment with drugs that exert protective effects on the vasculature does not alter the injurious nature of the exosomal properties elicited by IH. Instead, the beneficial role played by compounds such as CEL or LOS, appears to be mediated *via* a direct effect on the endothelium, while exosomes retain their biological properties for as long as IH persists. Indeed, as we have previously shown the effects of IH-exposed exosomes will abate within a few days after cessation of IH and return to baseline (10), further lending support to the direct effects of drugs on the endothelium rather than on the exosomal cargo.

This study employed several methodological approaches that deserve comment. First, the same subjects underwent identical IH exposures during the two runs and were randomized as far as the order of drug or P administration with sufficient time intervals allowed between each run, such as to prevent any potential residual effect of one exposure over the next. However, these participating subjects were healthy, and their IH exposures were relatively brief (6 h), such that the impact of either LOS or CEL on the vasculature in actual sleep apneic patients whose disease has usually been present for extended periods of time remains unclear. Thus, the effects of LOS or CEL on the cargo of circulating exosomes and on their functional properties are also unknown among sleep apnea patients, and will require randomized controlled trials to ascertain their potential contributions to the management of OSA, particularly when the latter is accompanied by the concurrent presence of cardiovascular morbidities. These considerations are of relevance in light of the findings reported by several studies in patients with OSA, whereby the beneficial effects of LOS on systemic blood pressure were less favorable than in patients with hypertension but without OSA (23–25). Accordingly, it has been postulated that the favorable effects of LOS may be partially mediated by their effects on the enhanced and sustained sympathoexcitation elicited by chronic IH (26). Of note, angiotensin receptor blockers appear to be more effective than CPAP alone in the reduction of systemic blood pressure among hypertensive patients with OSA (27). Such beneficial effects of LOS may not only represent the reductions in oxidative stress elicited by IH (8) but also the parallel attenuation of recruitment and propagation of inflammatory pathways in endothelium (28). In a previous study examining the effects of circulating microparticles collected from the plasma of patients with OSA, Tual-Chalot and colleagues reported that i.v. administration of such microparticles to naïve mice resulted in vascular hyperreactivity that exhibited both reduced eNOS and increased COX-2 dependencies (29). Thus, it is reasonable to assume that the beneficial effects of CEL *in vivo* in IH-exposed experimental humans may reflect the protective effects of COX-2 inhibition of the endothelium rather than specifically alter the composition of exosomes.

It is also noteworthy that although the contributory role of exosomes generated during IH exposures or in the context of OSA in inducing increased permeability and dysfunction of endothelial cells has now been established (10, 13), it has not yet been determined to be the controlling and determinant factor dictating endothelial cellular damage. With our current findings, there is evidence that exosomes are *de facto* effectors of endothelial dysfunction and that drug therapies can protect the endothelium from exosomal damage by their direct effects on the affected cells *via* their specific pharmacological attributes and targeted pathways.

## Conclusion

This study demonstrates that the functional properties and adverse of plasma exosomes generated and released to the circulation during IH remain unperturbed by concurrent treatment with either a COX-2 inhibitor or an angiotensin II receptor 1 antagonist. These findings enable us to interpret the beneficial effects of such pharmacological agents as reflecting their direct impact on the endothelium. It is possible that identification of specific miRNAs in exosomal cargo of OSA patients may enable delineation of additional therapeutic options specifically aimed at reducing the deleterious roles played by these extracellular vesicles in the vasculature of patients with OSA.

## Ethics Statement

This study was carried out in accordance with the recommendations of the Conjoint Health Research Ethics Board at the University of Calgary, and written informed consent was obtained from each subject before participating in the study. In addition the study was also approved by the Human Subject committee at the University of Chicago (protocol #: 10-702-A-CR004).

## Author Contributions

AK performed major components of experiments, analyzed data, and drafted initial version of the manuscript. NY, GF, AB, ZQ, VP, MP, and PH performed experiments, analyzed data, and contributed to editing of the manuscript. LK-G, MJP, and DG participated in the conceptualizing the study and edited the manuscript. All the authors have viewed and approved the final version of the manuscript.

## Conflict of Interest Statement

The authors declare that the research was conducted in the absence of any commercial or financial relationships that could be construed as a potential conflict of interest.

## Footnotes

^1^http://www.biophysics.com/products-ecisz0.php.

^2^http://www.essenbioscience.com/en/products/incucyte.

## Supplementary Material

Figure S1 Flow cytometry detection of surface molecules on exosomes derived from human subjects exposed to intermittent hypoxia (6 h/day) for 4 days. The data are graphed showing forward scatter versus FITC intensity. The first panel depicts beads with no exosomes then with exosomes. The degree of flow separation is shown on the right side for each capture set. The flow cytometry analysis of purified exosome following specific isolation with magnetic beads stained with anti-CD9, CD31, HLA-G, and Rab5b using a FACS analysis (FACSCalibur) instrument. The Exo-Flow magnetic stand for exosome separation and FACS analysis showing the absence of exosomes (negative, green color) and the presence of exosomes (positive, red color) beads are displayed on the FACS plot. The FITC flow cytometric intensities are then plotted versus the number of exosome particles input into the flow reaction (*n* = 6–8 for each antibody).

Figure S2 Examples of western blots as reported in Figure 5.

## Abbreviations

IH, intermittent hypoxia; CEL, celecoxib; LOS, losartan; P, placebo; OSA, obstructive sleep apnea; IH-LOS, IH + losartan; IH-P, IH + placebo; ECIS, electric cell-substrate impedance sensing; CLT, control; IH + P, intermittent hypoxia (IH) + placebo (P); IH + VEH (vehicle), intermittent hypoxia (IH) + VEH (vehicle); IH + CEL, intermittent hypoxia (IH)+ celecoxib (CEL).

## References

[1] Beaudin AE Waltz X Hanly PJ Poulin MJ. Impact of obstructive sleep apnoea and intermittent hypoxia on cardiovascular and cerebrovascular regulation. Exp Physiol (2017) 102:743–63. 10.1113/EP086051 28439921

[2] Javaheri S Barbe F Campos-Rodriguez F Dempsey JA Khayat R Javaheri S Sleep apnea: types, mechanisms, and clinical cardiovascular consequences. J Am Coll Cardiol (2017) 69:841–58. 10.1016/j.jacc.2016.11.069 28209226 PMC5393905

[3] Eichhorn L Dolscheid-Pommerich R Erdfelder F Ayub MA Schmitz T Werner N Sustained apnea induces endothelial activation. Clin Cardiol (2017) 40:704–9. 10.1002/clc.22720 28464406 PMC6490346

[4] Tremblay JC Boulet LM Tymko MM Foster GE. Intermittent hypoxia and arterial blood pressure control in humans: role of the peripheral vasculature and carotid baroreflex. Am J Physiol Heart Circ Physiol (2016) 311:H699–706. 10.1152/ajpheart.00388.2016 27402667

[5] Smith SM Friedle SA Watters JJ. Chronic intermittent hypoxia exerts CNS region-specific effects on rat microglial inflammatory and TLR4 gene expression. PLoS One (2013) 8:e81584. 10.1371/journal.pone.0081584 24324707 PMC3852519

[6] Ren J Liu W Deng Y Li GC Pan YY Xie S Losartan attenuates aortic endothelial apoptosis induced by chronic intermittent hypoxia partly via the phospholipase C pathway. Sleep Breath (2017) 21:679–89. 10.1007/s11325-017-1479-4 28190165

[7] Foster GE Hanly PJ Ahmed SB Beaudin AE Pialoux V Poulin MJ. Intermittent hypoxia increases arterial blood pressure in humans through a renin-angiotensin system-dependent mechanism. Hypertension (2010) 56:369–77. 10.1161/HYPERTENSIONAHA.110.152108 20625082

[8] Pialoux V Foster GE Ahmed SB Beaudin AE Hanly PJ Poulin MJ. Losartan abolishes oxidative stress induced by intermittent hypoxia in humans. J Physiol (2011) 589:5529–37. 10.1113/jphysiol.2011.218156 21930596 PMC3240889

[9] Su SA Xie Y Fu Z Wang Y Wang JA Xiang M. Emerging role of exosome-mediated intercellular communication in vascular remodeling. Oncotarget (2017) 8:25700–12. 10.18632/oncotarget.14878 28147325 PMC5421963

[10] Khalyfa A Zhang C Khalyfa AA Foster GE Beaudin AE Andrade J Effect on intermittent hypoxia on plasma exosomal micro RNA signature and endothelial function in healthy adults. Sleep (2016) 39:2077–90. 10.5665/sleep.6302 27634792 PMC5103796

[11] Beaudin AE Pun M Yang C Nicholl DD Steinback CD Slater DM Cyclooxygenases 1 and 2 differentially regulate blood pressure and cerebrovascular responses to acute and chronic intermittent hypoxia: implications for sleep apnea. J Am Heart Assoc (2014) 3:e000875. 10.1161/JAHA.114.000875 24815497 PMC4309085

[12] Beaudin AE Waltz X Pun M Wynne-Edwards KE Ahmed SB Anderson TJ Human intermittent hypoxia-induced respiratory plasticity is not caused by inflammation. Eur Respir J (2015) 46:1072–83. 10.1183/09031936.00007415 26065565

[13] Khalyfa A Kheirandish-Gozal L Khalyfa AA Philby MF Alonso-Alvarez ML Mohammadi M Circulating plasma extracellular microvesicle microRNA cargo and endothelial dysfunction in children with obstructive sleep apnea. Am J Respir Crit Care Med (2016) 194:1116–26. 10.1164/rccm.201602-0323OC 27163713 PMC5114451

[14] Khalyfa A Almendros I Gileles-Hillel A Akbarpour M Trzepizur W Mokhlesi B Circulating exosomes potentiate tumor malignant properties in a mouse model of chronic sleep fragmentation. Oncotarget (2016) 7:54676–90. 10.18632/oncotarget.10578 27419627 PMC5342372

[15] Khalyfa A Khalyfa AA Akbarpour M Connes P Romana M Lapping-Carr G Extracellular microvesicle microRNAs in children with sickle cell anaemia with divergent clinical phenotypes. Br J Haematol (2016) 174:786–98. 10.1111/bjh.14104 27161653

[16] Lotvall J Hill AF Hochberg F Buzas EI Di Vizio D Gardiner C Minimal experimental requirements for definition of extracellular vesicles and their functions: a position statement from the International Society for Extracellular Vesicles. J Extracell Vesicles (2014) 3:26913. 10.3402/jev.v3.26913 25536934 PMC4275645

[17] Tiruppathi C Malik AB Del Vecchio PJ Keese CR Giaever I. Electrical method for detection of endothelial cell shape change in real time: assessment of endothelial barrier function. Proc Natl Acad Sci U S A (1992) 89:7919–23. 10.1073/pnas.89.17.7919 1518814 PMC49826

[18] Pitt JM Kroemer G Zitvogel L. Extracellular vesicles: masters of intercellular communication and potential clinical interventions. J Clin Invest (2016) 126:1139–43. 10.1172/JCI87316 27035805 PMC4811136

[19] Tkach M Thery C. Communication by extracellular vesicles: where we are and where we need to go. Cell (2016) 164:1226–32. 10.1016/j.cell.2016.01.043 26967288

[20] Fevrier B Vilette D Archer F Loew D Faigle W Vidal M Cells release prions in association with exosomes. Proc Natl Acad Sci U S A (2004) 101:9683–8. 10.1073/pnas.0308413101 15210972 PMC470735

[21] Stoorvogel W Kleijmeer MJ Geuze HJ Raposo G. The biogenesis and functions of exosomes. Traffic (2002) 3:321–30. 10.1034/j.1600-0854.2002.30502.x 11967126

[22] van Niel G Porto-Carreiro I Simoes S Raposo G. Exosomes: a common pathway for a specialized function. J Biochem (2006) 140:13–21. 10.1093/jb/mvj128 16877764

[23] Kraiczi H Hedner J Peker Y Grote L. Comparison of atenolol, amlodipine, enalapril, hydrochlorothiazide, and losartan for antihypertensive treatment in patients with obstructive sleep apnea. Am J Respir Crit Care Med (2000) 161:1423–8. 10.1164/ajrccm.161.5.9909024 10806134

[24] Thunstrom E Manhem K Yucel-Lindberg T Rosengren A Lindberg C Peker Y Neuroendocrine and inflammatory responses to losartan and continuous positive airway pressure in patients with hypertension and obstructive sleep apnea. A randomized controlled trial. Ann Am Thorac Soc (2016) 13:2002–11. 10.1513/AnnalsATS.201602-126OC 27548072

[25] Thunstrom E Manhem K Rosengren A Peker Y. Blood pressure response to losartan and continuous positive airway pressure in hypertension and obstructive sleep apnea. Am J Respir Crit Care Med (2016) 193:310–20. 10.1164/rccm.201505-0998OC 26414380

[26] Fenik VB Singletary T Branconi JL Davies RO Kubin L. Glucoregulatory consequences and cardiorespiratory parameters in rats exposed to chronic-intermittent hypoxia: effects of the duration of exposure and losartan. Front Neurol (2012) 3:51. 10.3389/fneur.2012.00051 22509173 PMC3321439

[27] Pepin JL Tamisier R Barone-Rochette G Launois SH Levy P Baguet JP. Comparison of continuous positive airway pressure and valsartan in hypertensive patients with sleep apnea. Am J Respir Crit Care Med (2010) 182:954–60. 10.1164/rccm.200912-1803OC 20522795

[28] Fliser D Buchholz K Haller H EUropean Trial on Olmesartan and Pravastatin in Inflammation and Atherosclerosis (EUTOPIA) Investigators. Antiinflammatory effects of angiotensin II subtype 1 receptor blockade in hypertensive patients with microinflammation. Circulation (2004) 110:1103–7. 10.1161/01.CIR.0000140265.21608.8E 15313950

[29] Tual-Chalot S Fatoumata K Priou P Trzepizur W Gaceb A Contreras C Circulating microparticles from patients with obstructive sleep apnea enhance vascular contraction: mandatory role of the endothelium. Am J Pathol (2012) 181:1473–82. 10.1016/j.ajpath.2012.06.020 22846722

