# Supplementary figures and images for: Plasma Exosomes and Improvements in Endothelial Function by Angiotensin 2 Type 1 Receptor or Cyclooxygenase 2 Blockade following Intermittent Hypoxia
Source: Front Neurol. 2017 Dec 22;8:709. doi: 10.3389/fneur.2017.00709 (PMC5743928; doi:10.3389/fneur.2017.00709)

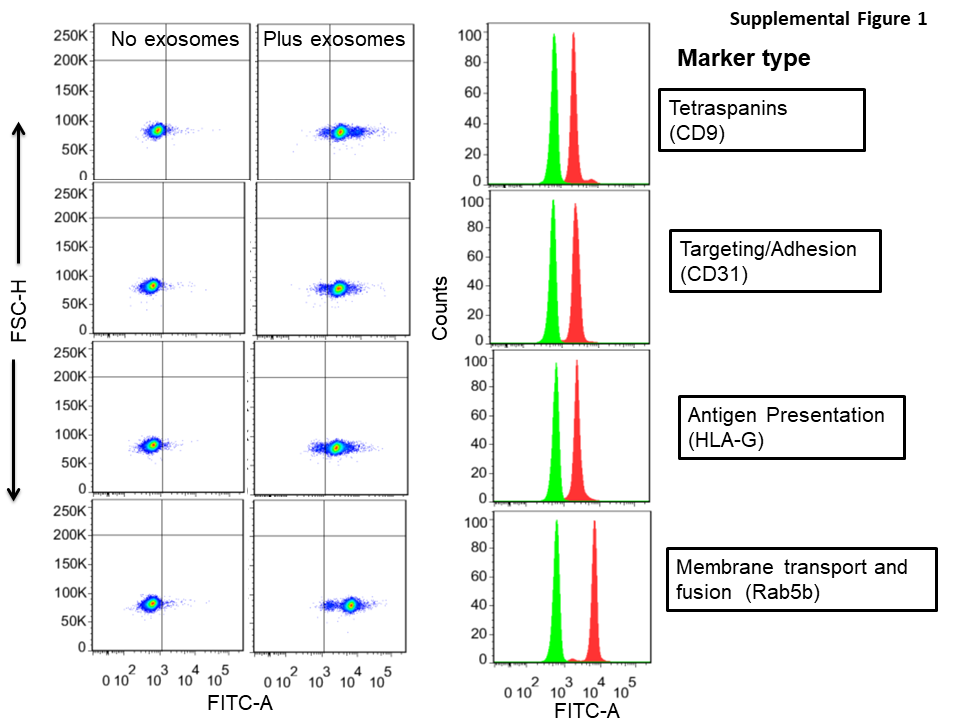

Supplement: Figure S1 — Flow cytometry detection of surface molecules on exosomes derived from human subjects exposed to intermittent hypoxia (6 h/day) for 4 days. The data are graphed showing forward scatter versus FITC intensity. The first panel depicts beads with no exosomes then with exosomes. The degree of flow separation is shown on the right side for each capture set. The flow cytometry analysis of purified exosome following specific isolation with magnetic beads stained with anti-CD9, CD31, HLA-G, and Rab5b using a FACS analysis (FACSCalibur) instrument. The Exo-Flow magnetic stand for exosome separation and FACS analysis showing the absence of exosomes (negative, green color) and the presence of exosomes (positive, red color) beads are displayed on the FACS plot. The FITC flow cytometric intensities are then plotted versus the number of exosome particles input into the flow reaction (n = 6–8 for each antibody). [file Image_1.TIF]

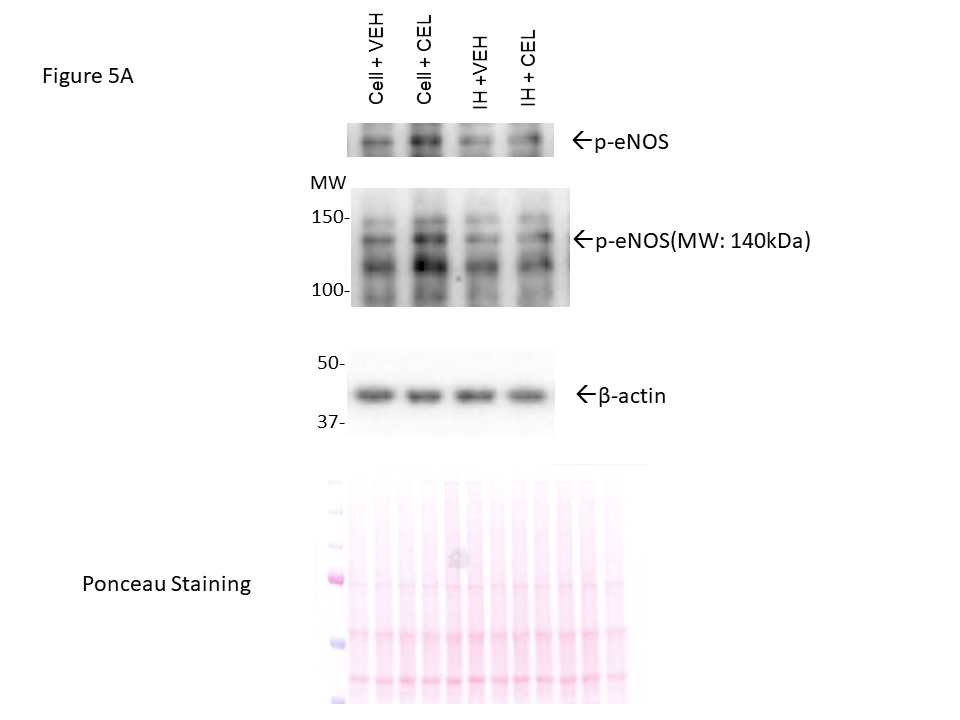

Supplement: Figure S2 — Examples of western blots as reported in Figure 5. [file Image_2.TIF]

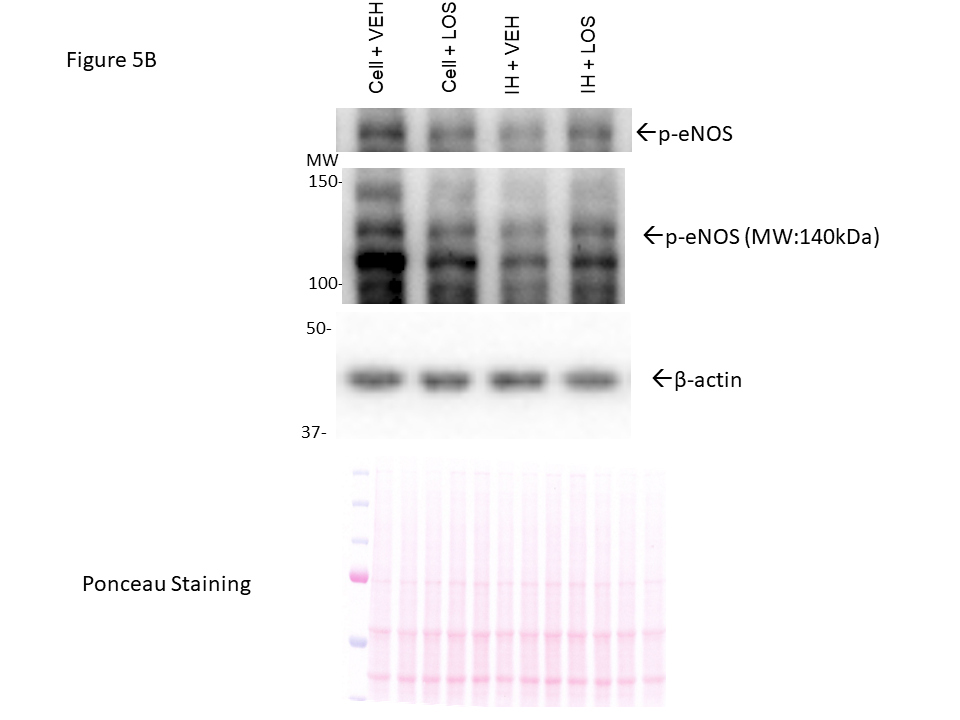

Supplement: Supplementary file 3 [file Image_3.TIF]

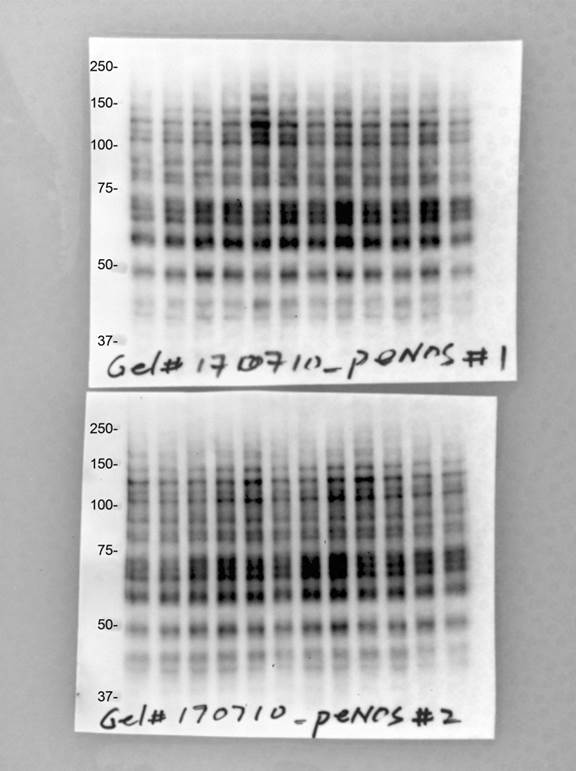

Supplement: Supplementary file 4 [file Image_4.JPEG]
